# Natural Compounds as Modulators of NADPH Oxidases

**DOI:** 10.1155/2013/271602

**Published:** 2013-11-27

**Authors:** Tullia Maraldi

**Affiliations:** Department of Surgical, Medical, Dental and Morphological Sciences with Interest in Transplant, Oncology and Regenerative Medicine, University of Modena and Reggio Emilia, Via Del Pozzo 71, 41100 Modena, Italy

## Abstract

Reactive oxygen species (ROS) are cellular signals generated ubiquitously by all mammalian cells, but their relative unbalance triggers also diseases through intracellular damage to DNA, RNA, proteins, and lipids. NADPH oxidases (NOX) are the only known enzyme family with the sole function to produce ROS. The NOX physiological functions concern host defence, cellular signaling, regulation of gene expression, and cell differentiation. On the other hand, increased NOX activity contributes to a wide range of pathological processes, including cardiovascular diseases, neurodegeneration, organ failure, and cancer. Therefore targeting these enzymatic ROS sources by natural compounds, without affecting the physiological redox state, may be an important tool. This review summarizes the current state of knowledge of the role of NOX enzymes in physiology and pathology and provides an overview of the currently available NADPH oxidase inhibitors derived from natural extracts such as polyphenols.

## 1. ROS Involvement in Cell Pathophysiology

Oxidative stress is a molecular deregulation in reactive oxygen species (ROS) metabolism involved in the pathogenesis of several diseases. Oxidative stress is no longer considered as a simple imbalance between the production and scavenging of ROS, but as a dysfunction of enzymes involved in ROS production [1].

Reactive oxygen species such as superoxide, hydrogen peroxide, and peroxynitrite are generated by all mammalian cells and have been recognized for many decades as causing cell damage by oxidation and nitration of macromolecules, such as DNA, RNA, proteins, and lipids. Moreover, ROS can also promote cell signaling pathways modulated by growth factors and transcription factors, therefore regulating cell proliferation, differentiation, and apoptosis [2], which are important processes for proper cell functioning [3]. At physiological concentrations they facilitate the signal transduction derived from receptor tyrosine kinases and transcriptional factors such as NF-E2-related factor-2 (Nrf-2) leading to antioxidant gene expression [4].

The instability of an unpaired electron in its valence shell causes the high reactivity of superoxide. Superoxide has been implicated in numerous pathological processes, including cancer, cardiovascular disease (e.g., atherosclerosis and stroke), and acute and chronic diseases due to microbial infections. Superoxide can directly or indirectly damage DNA through oxidation [5], directly inactivate cellular antioxidants enzymes such as catalase and glutathione peroxidase [6], and activate proinflammatory nuclear factor jB (NF-jB) [7].

However, superoxide gives rise to other ROS that possess different redox chemistries, and, thus, different physiological and pathophysiological effects. For example, superoxide is rapidly reduced, both spontaneously and enzymatically, to H_2_O_2_. Unlike superoxide, H_2_O_2_ has no net charge; so, it is more lipid-soluble, with the potential to diffuse through organelles and cellular membranes reaching sites distant from its source. H_2_O_2_ modifies cellular proteins via oxidation of cysteine, methionine [8], and genetic material [9]. However, perhaps the major dangerous properties of H_2_O_2_ are in its ability to generate more reactive molecules. For instance, in the presence of transition metals, H_2_O_2_ can generate the highly reactive OH•. The OH• is highly reactive and will indiscriminately oxidize the nucleotides causing breaks and lesions of DNA [for review see [10]], which are processes involved in carcinogenesis. The oxidation of lipids by OH• may influence many physiological processes and contribute to cellular dysfunction, such as oxidation of lipids by peroxidation [11], during cardiovascular disease [12].

One of the most important and fast redox reactions in biology is between superoxide and the nitric oxide (NO) radical giving rise to ONOO^−^. ONOO^−^ is an oxidizing and nitrating molecule that has been implicated in cancer [13] and other acute [14] and chronic [15] diseases. ROS levels in tumor cells are controlled in a particular way, which stresses the importance of the development of novel ROS-targeted anticancer therapies.

As with every mechanism involved in both normal cell function and the development of disease, strategies to counteract ROS must take into account their critical importance in the normal functioning of the organism [1].

Further understanding of the biological mechanisms among oxidative stress, tumor growth, and metastasis could contribute to the advancement of cancer treatment.

For example, angiogenesis is another important factor for tumor growth and metastasis, and ROS has a key role in angiogenesis regulation [16].

After all, an emerging concept suggests that ROS modulate the immune cells functions that infiltrate the tumor environment and stimulate angiogenesis [2].

These oxidative processes have been implicated in many diseases in addition to cancer.

Overproduction of ROS is involved in the development of a number of diseases, which range from neurological such as Parkinson's [17] and Alzheimer's disease [18], to psychiatric disorders such as schizophrenia [19] and bipolar disorder [20], and to a majority of cardiovascular diseases [21].

## 2. NADPH Oxidases as ROS Sources

Several enzymes produce ROS, including the mitochondrial electron transport chain, nitric oxide synthases (NOSs), cytochrome P450 reductase, and xanthine oxidase. However, for all of these systems, ROS production takes place as a byproduct of the main catalytic function of the enzyme/system or from a dysfunctional variant of the enzyme. In contrast, NADPH oxidases are the only enzymes whose primary function is to generate superoxide/ROS [2]. NOX family proteins are the catalytic, electrontransporting subunits of the NADPH oxidase enzyme complex. The NOX family consists of seven members, NOX1–5, and two dual oxidases (Duox), Duox1 and Duox2 (Table 1). The NOX isoforms contain FAD and NADPH binding sites, two heme molecules and six transmembrane [22] spanning alpha helices with cytosolic N and C-termini. The Duox isoforms also contain the same domains; however, a seventh transmembrane domain and peroxidase homology are present.

They are differentially expressed and regulated in various tissues and have different subcellular localizations (reviewed in [23]). NOX1, NOX2, and NOX5 appear to produce mainly superoxide NOX4, mainly H_2_O_2_ [24]. All NOX isoforms have been reported to bind to one or more additional components. p22phox appears to be a general binding partner for NOX1–4. NOX1 and 2 also bind the small GTPase, Rac. Moreover, NOX1 binds the cytosolic subunits, NOX organizer 1 (NOXO1), and NOX activator 1 (NOXA1). NOX2 binds the respective homologues, p47phox and p67phox, and also the cytosolic protein, p40phox [22, 25]. NOX4 was reported to bind to the polymerase (DNA-directed) delta-interacting protein 2 (PolDip2) [26].

In addition to these established NOX binding partners, the tyrosine kinase substrate with 4/5 SH3 domains (Tks4/5) [27] and protein disulfide isomerase (PDI) were recently suggested to bind to both NOX1 and 4 [28]. Two maturation factors specific for Duox (DuoxA1 and DuoxA2) have also been described [22].

NOX catalytic subunits are differently regulated: NOXA1 plays a key role for NOX1 activation [29], p67phox for NOX2 [30], and calmodulin for NOX5 [31].

In contrast, NOX4 is constitutively active, and modulation of its expression may thus be a major activity regulator.

ROS produced by NOXs have been shown to affect all other possible sources of ROS, leading to their dysfunction and to a further increase in ROS generation, forming a vicious cycle of oxidative stress. For example, increased O_2_^•−^ generation by NADPH oxidase induces mitochondrial oxidative damage via structural changes to the inner mitochondrial membrane and disturbs flow in the electron transport chain which enhances ROS production [32].

## 3. NOX and Pathophysiology

The oxidant signaling involving NADPH oxidase has important roles in cell biology participating in intracellular signaling of cell differentiation and proliferation. These mechanisms are important in tissue repair and tumorigenesis, processes where cell proliferation occurs, but when poorly controlled the generation of ROS is dangerous. Indeed, NADPH oxidase-mediated cell proliferation has been observed in a wide range of cell types including those found in blood vessels, kidney, liver, skeletal muscle precursors, neonatal cardiac myocytes, lung epithelial cells, gastric mucosa, brain microglia, and a variety of cancer cells. For example, NOX may stimulate Akt activation also by inactivating the phosphatase PTEN, a direct negative regulator of the PI3K/Akt pathway [33]. Therefore, NADPH oxidase-mediated redox signaling may amplify diverse signaling pathways triggered in tissue repair processes such as cell proliferation, wound healing, angiogenesis, or fibrosis. Recent studies also suggest that NADPH oxidase is involved in differentiation and proliferation of stem cells. Currently, little is known about whether ROS regulate different signaling pathways in stem cells and differentiated cells and whether ROS play a different role in these cells [16]. Thus, modulating NADPH oxidase may have significant impacts on regenerative medicine and tissue engineering, such as growing heart muscle.

At first, cellular stresses may induce NOX-dependent ROS generation as an alert system that drives the cells into a relatively stress-resistant status by integrating and amplifying the stress signals, as preconditioning against further cellular challenges [34]. The double-edged sword property of NOX adds another level of complexity for therapeutic targeting of this enzyme. The mechanisms of NOX-mediated redox signaling may be either enhancing stress resistance through prevention of ischemia-reperfusion injuries and accelerating wound healing, or avoiding the stress-induced cytotoxicity for iper-proliferative diseases, such as cancer. Therefore, a therapeutic modulation of NOX activity has to be developed taking in account the disease, the stage of disease, the tissue localization area, the NOX isoforms, and the NOX intracellular localization.

## 4. NOXs and Diseases

NADPH oxidase has received much attention as a major cause of oxidative stress leading to vascular disease. Moreover different NOX subunits have been suggested to play a role in cancer, lung fibrosis, stroke, heart failure, diabetes, and neurodegenerative diseases [22]. NOX-derived ROS can lead to pathology through differential ways: for example, spatially confined levels of ROS that interfere with a particular signaling pathway, and high levels (local or systemic) that are directly cytotoxic, causing apoptosis, or disturbing redox-sensitive signaling cascades.

### 4.1. Vascular Diseases

NADPH oxidases are ubiquitously expressed in tissues but are the major source of superoxide anions observed in the vasculature [1]. Thus, similarly to ROS, NOX proteins have both beneficial and harmful effects. They are important signaling molecules which regulate vascular tone, expression, proliferation, migration, and differentiation. On the other hand, cardiovascular risk factors and vascular diseases increase ROS and contribute to atherosclerosis, vascular dysfunction, hypertension, vascular hypertrophy, and thrombosis.

With respect to low and spatially confined ROS overproduction, NOX1 is a good candidate to migrate into caveolae and there causes eNOS uncoupling and endothelial dysfunction, which is often associated with increased blood pressure and enhanced platelet aggregation as an early step in the development of atherosclerosis [35].

### 4.2. Cancer

NOX1, NOX2, and NOX4 are known to be expressed in multiple tumor cell types (see Table 1). Tumor cells produce high amounts of ROS [36, 37] by NADPH oxidases to promote their own proliferation, through regulation of proliferative signaling kinases, such as cell survival factors, such as Akt and MEK-ERK pathway [38].

Recently, the role of cancer stem cells (CSCs) in cancer progression and metastasis has attracted much attention since CSCs are integral parts of pathophysiologic mechanisms of metastasis and chemo/radioresistance [16]. To date, molecular events that govern the survival and self-renewal of CSCs are poorly defined, but a link between ROS modulation and cancer stem cell metabolism seems to have a basis. Stem cells and also CSCs are known to reside in niches characterized by low ROS, a critical factor in maintaining stem cell properties such as self-renewal. Multiple signaling pathways in normal stem cells and CSCs can regulate ROS level and could be exploited against CSCs. Elucidation of ROS function in CSCs will enrich our knowledge of cancer development and metastasis.

Moreover, ROS have also been shown to regulate angiogenesis through the release and actions of tumor-derived growth factors that induce endothelial cell proliferation. In fact, ROS production within tumor cells dramatically promotes the release of paracrine growth factors such as VEGF and the expression of its receptor, VEGF receptor-1 which, in turn, stimulate proliferation, migration, and tube formation in nearby endothelial cells [34]. NADPH oxidase within endothelial cells cooperates with growth factors, such as VEGF released by tumors, to stimulate endothelial cell proliferation and then angiogenesis. Thus, NOXs as ROS source in tumors and in endothelium may be considered novel targets for antiangiogenic treatment.

### 4.3. Inflammation

The oxidative burst of phagocytes has long been considered proinflammatory, causing cell and tissue destruction. Recent findings have challenged this inflammatory role of ROS, and now ROS are also known to regulate immune responses and cell proliferation and to determine T-cell autoreactivity. NOX2-derived ROS have been shown to suppress antigen-dependent T-cell reactivity and remarkably to reduce the severity of experimental arthritis in both rats and mice [39]. In fact, regarding these chronic inflammatory diseases (rheumatoid arthritis) there is increasing evidence that ROS can often help to modulate inflammation, ensuring that it does not become too prolonged [see review [40]].

In retrospect, this is also suggested by the pathology of chronic granulomatous disease (a condition characterized by inborn defects in the phagocyte O_2_^•−^ generating NADPH oxidase), there is an increased risk of infection due to an inability to kill certain microorganisms; this is in addition to a severe dysregulation and prolongation of inflammation. Thus, stopping ROS production can be deleterious. On the other hand, ROS can cause severe cartilage damage and the ability of nuclear factor erythroid 2 p4-5-related factor 2 to enhance endogenous antioxidant defenses in response to the inflammation may play a significant ameliorative role. The same may be true in sepsis; some ROS may help but too many can cause harm [see review [40]].

Finally, NADPH oxidase-derived ROS are also crucial players of tumor anti-immunity regulating specialized subsets of immune cells such as macrophages and T lymphocytes. Thus, NOXs could represent a novel molecular link between chronic inflammation and angiogenesis during cancer [2].

NOX2 is connected to the innate immune response [41], including to fungal infections [42] and adaptive immune response at the level of both T cells and antigen-presenting cells [43]. Thus, NOX2 inhibition leads to an improvement of diseases involving a significant inflammatory response. On the other hand, the essential immune functions of NOX2 have to be preserved [35].

### 4.4. Organ Failure

ROS are generally thought to play an important role in the pathophysiology of organ failure [22]. For example, in liver and intestinal tissue injury, there is some indication for a pathogenetic role of NOX2-derived ROS by neutrophils [44, 45].

With respect to high levels of ROS produced by NOX4, they can be directly cytotoxic or cause apoptosis inducing heart ischemic stroke. On the other hand, regarding the NOX4 role on pressure overload of the heart, NOX4 might be responsible of both acute damage of the cardiomyocyte and subacute protection of the heart by promoting angiogenesis [35].

### 4.5. CNS Diseases

#### 4.5.1. Ischemic Stroke

In a gerbil model of global cerebral ischemia-reperfusion injury, NOX inhibition by apocynin strongly diminishes damage to the hippocampus [46]. Stroke size was markedly reduced in NOX2-deficient mice [47], while increased NOX2 expression in diabetic rats was associated with an aggravated ischemic brain injury [48].

#### 4.5.2. Alzheimer's Disease, Parkinson's Disease, and HIV Dementia

NOX2 seems to have a role in inflammatory neurodegeneration diseases, including Alzheimer's disease and Parkinson's disease [49, 50]. In the case of Alzheimer's disease, amyloid precursor protein fragments released from neurons activate NOX2 -dependent ROS generation by microglia cells leading to death of neighboring neurons [51]. Several studies suggest similar mechanisms, involving NOX2, in Parkinson's disease [52, 53].

Microglia activation is also thought to be a key element in the development of dementia [54], and a role of NOX2 activation in animal model of dementia has been suggested [55].

Microglial NOX2-derived ROS have also been implicated in the progression of the demyelinating disease through phagocytosis of myelin and damage to the myelin sheath [56]. In periventricular leukomalacia, the combination of NOX2-derived superoxide and inducible nitric oxide synthase-derived nitric oxide leads to the formation of peroxynitrate and thereby to the killing of oligodendrites [57].

## 5. Antioxidant Therapy

Classically, oxidative stress has been defined as an imbalance between the endogenous production of reactive oxygen compounds and the antioxidative potential of cells [58].

But the low or apparent lack of clinical effectiveness of ROS-scavenging approaches is not entirely explained. It can be due to the partial removal of selected harmful endproducts by ROS-scavengers. Furthermore, antioxidants, including vitamins, reaction with superoxide anions is slower than NO. Moreover, it does not take into account that cellular events leading to disease primarily occur in individual cellular compartments [3].

### 5.1. NOX Inhibition (See Table 2)

The main sources of ROS include redox enzymes such as the respiratory chain, xanthine oxidase, lipooxygenase, cyclooxygenase, and NADPH oxidases, and these systems are continuously interacting with each other. Due to the complex mechanisms involved in the activation of NADPH oxidases, these enzymes can be targeted on several different levels of their activity. Firstly, decreasing NADPH oxidase expression can inhibit them. Also, the activation of NADPH oxidase can be decreased by blocking the translocation of its cytosolic subunits, when present, to the membrane.

Another possibility is inhibition of the p47phox subunit, either by preventing its phosphorylation using PKC inhibitors or by blocking its binding to other subunits. A decrease of signal transduction and inhibition of Rac 1 translocation has also been demonstrated to decrease ROS generation [3].

Several compounds have been used, including apocynin, diphenylene iodonium (DPI), and 4-(2-aminoethyl)-benzensulfonylfluorid (AEBSF). However, it has become apparent that these inhibitors are not specific for NOX [59] and not selective for single NOX isoforms.

One of the first inhibitors used in model studies was diphenyliodonium (DPI), which is very potent (although in micromolar range) but lacks specificity. DPI is a general flavoprotein inhibitor, also inhibiting, for example, xanthine oxidase and eNOS [60–62], as well as cholinesterases and a calcium pump [63].

AEBSF is primarily a serine protease inhibitor [64]. Its greatest drawback is however toxicity.

Later studies involved apocynin, a naturally occurring NADPH oxidase inhibitor originally isolated from the roots of Picrorhiza kurroa. Apocynin cannot be used as selective NADPH oxidase inhibitor due to its direct antioxidant and several off-target effects [60]. Apocynin is an orally active agent that can block NADPH oxidase assembly in membrane but requires a reaction with peroxidase for its activation, and therefore does not work immediately [65]. Apocynin reduces ROS production in models of arthritis, asthma, and hypertension, abolishing the increase in vascular O_2_^•−^ and preventing endothelial dysfunction [66]. However, effects of apocynin are not specific, as it has been reported to affect arachidonic acid metabolism [67], to increase glutathione synthesis, and to activate the AP-1 transcription factor [68]. In addition, it has recently been shown to be a direct ROS scavenger in certain experimental conditions [69].

The use of natural antioxidants represents a promising new approach for NOX inhibition. Polyphenols represent more than 10000 compounds occurring naturally in foods and the recommendation for a polyphenol rich (green tea/red wine/fruits/vegetables/whole grain foods) diet in the prevention of cardiovascular disease is still valid [70]. It was found that polyphenols, apart from their well-known superoxide radical scavenging abilities, decrease NADPH oxidase activity [71] in a number of tissues including vessels and platelets [72]. Moreover, novel polyphenolic compounds are being investigated which lack typical superoxide scavenging properties and directly inhibit NADPH oxidase.

Recent studies with berberine, a plant alkaloid [73], revealed an inhibition of NADPH oxidase activity and reduction of gp91phox mRNA expression in macrophages. Also, emodin, an active component extracted from rhubarb and rhein, reduced ROS generation [74]. Similar effects were observed by treatment with 3-(4′-hydroxyl-3′,5′-dimethoxyphenyl)propionic acid (HDMPPA), the active ingredient in kimchi, ellagic acid, a polyphenol present in fruits and nuts [75], and dihomo-*γ*-linolenic (*ω*-6) acid [76].

The inhibitory effect of flavonoids (kaempferol, morin, quercetin, and fisetin) on the respiratory burst of neutrophils was observed by Pagonis et al. [77] as early as in 1986.

A Ginkgo biloba extract containing flavonoids, among other compounds, was tested by Pincemail et al. [78] for its effect on the release of ROS (superoxide anion radical, hydrogen peroxide, and hydroxyl radical) during the stimulation of human neutrophils by a soluble agonist. The extract slowed down the oxygen consumption (respiratory burst) of the stimulated cells by its inhibitory action on NADPH oxidase [79]. The extract was also able to reduce the activity of myeloperoxidase contained in neutrophils. Moreover, it had free radical scavenging activity.

A higher number of hydroxyl substituents are an important structural feature of flavonoids in respect to their scavenging activity against ROS, while C-2,3 double bond (present in quercetin and resveratrol) might be important for the inhibition of ROS production by phagocytes [80].

The bark of magnolia has been used in oriental medicine to treat a variety of remedies, including some neurological disorders [81]. Magnolol (Mag) and honokiol (Hon) are isomers of polyphenolic compounds from the bark of Magnolia officinalis, and have been identified as major active components exhibiting antioxidative, anti-inflammatory, and neuroprotective effects. It has been reported that exposure of Hon and Mag to neurons for 24 h did not alter neuronal viability, but both compounds inhibited superoxide production, a pathway known to involve NADPH oxidase. This study highlighted the important role of NADPH oxidase in mediating oxidative stress in neurons and microglial cells and has unveiled the role of IFN*γ* in stimulating the MAPK/ERK1/2 signaling pathway for activation of NADPH oxidase in microglial cells. Hon and Mag offer anti-oxidative or anti-inflammatory effects, at least in part, through suppressing IFN*γ*-induced p-ERK1/2 and its downstream pathway [81].

Incubation of human neuroblastoma cells with nonpolar blueberry fractions obstructed the coalescing of lipid rafts into large domains disrupting NOX assembly therein and abolishing ROS production [82]. In fact, this NOX inhibiting bioactivity in crude blueberry extracts partitioned into a polyphenol-devoid fraction lacking virtually any antioxidant capacity and prevented proper assembly of the multisubunit NOX complex interfering with the coalescence of large lipid raft domains.

Prodigiosin, a microbial pigment, and some derivatives suppressed NOX activity most likely by disrupting Rac function [83, 84], and inhibition of NOX by the alkaloid sinomenine is unclear at best [85].

In an oxygen-glucose deprivation and reoxygenation (OGD/R) model, pretreatment with green tea polyphenols (GTPP) and their active ingredient, epigallocatechin-3-gallate (EGCG), protects PC12 cells from subsequent OGD/R-induced cell death [86, 87]. GTPP stimulates laminin receptor and thereby induces NADPH oxidase-dependent generation of ROS, which in turn induces activation of PKC resulting in preconditioning against cell death induced by OGD/R.

Resveratrol is a naturally occurring polyphenol, which has vasoprotective effects in diabetic animal models and inhibits high glucose (HG-) induced oxidative stress in endothelial cells. It has been reported that HG induces endothelial cell apoptosis through NF-*κ*B/NADPH oxidase/ROS pathway, which was inhibited by resveratrol [88, 89].

Other NOX inhibitors are VAS 2870, VAS 3947, GK-136901, plumbagin, and polyphenolic derivative S17834 [90].

Plumbagin, a plant-derived naphthoquinone, has been shown to exert anticarcinogenic and antiatherosclerosis effects in animals. Plumbagin inhibits NADPH-dependent superoxide production in cell lines that express NOX4 oxidase [91]. Although its exact mechanism of action remains unclear, the inclusion of a napthoquinone structure within the molecule may be responsible for its ROS scavenging abilities [90]. Plumbagin inhibited the activity of NOX4 in a time- and dose-dependent manner in HEK293 and LN229 cells directly interacting with NOX4 and inhibiting its activity [91].

Indeed, plumbagin has been reported to exert anticancer activity on osteosarcoma cells by inducing proapoptotic signaling and modulating the intracellular ROS that causes induction of apoptosis [92]. Moreover plumbagin-induced AMPK activation might be the key mediator of plumbagin's antitumor activity [93].

Furthermore, PI5K-1B plays a crucial role in ROS generation and could be a new molecular target of plumbagin [94]. At last, plumbagin can exert its function in depleting glutathione (GSH) levels that led to increase in ROS generation [95].

Celastrol is one of several bioactive compounds extracted from the medicinal plant Tripterygium wilfordii. Celastrol is used to treat inflammatory conditions and shows benefits in models of neurodegenerative disease, cancer, and arthritis, although its mechanism of action is incompletely understood. Authors demonstrated that celastrol is a potent inhibitor of NOX enzymes in general with increased potency against NOX1 and NOX2. Furthermore, inhibition of NOX1 and NOX2 was mediated via a novel mode of action, namely, inhibition of a functional association between cytosolic subunits and the membrane flavocytochrome [96].

## 6. Conclusions

Accumulating evidence clearly indicates that NADPH oxidases are critical molecular targets for dietary bioactive agents for prevention and therapy of different pathologies.

The development of specific and not toxic inhibitors of NADPH oxidases and their redox signaling network (kinase, transcription factors, and genes) could provide useful therapeutic strategies for the treatment of oxidative stress dependent processes such as cancer and other degenerative diseases.

In fact, classical antioxidant therapies have been demonstrated inadequate since the importance of ROS in physiology has been ignored leading to the lack of clinical benefits. Indeed, further research into selective molecular inhibitors interfering with NADPH oxidase activation are warranted. The selective targeting of dysfunctional NADPH oxidase homologs appears to be the most suitable approach, with the potential to be far more efficient than the one with nonselective antioxidants having only ROS scavenging properties.

NOX enzymes, however, are very complex with numerous specific targets within each isoform. More information is needed on how these proteins are targeted to different subcellular compartments and how this transport process is regulated.

It is encouraging, however, that single bioactive dietary agents can directly and indirectly influence most, if not all, of the myriad targets within NOX family. Additionally, many of these dietary agents appear to exhibit some degree of specificity for redox deregulated cells while unaffecting normal cells balance. Moreover, the protective effects of some single agents could be potentiated and/or synergized by other dietary agents. While encouraging, there are many considerations that remain, such as the issue of appropriate dose of each agent, appropriate timing and duration of exposure, importance of cell type specificity, relative bioavailability of each agent, and potentially adverse side effects and interactions.

## Figures and Tables

**Table 1:** NOX isoforms and pathology [modified from [22, 23]].

Characteristic	Binding partners	Intracellular localization	Tissue distribution	Implication in pathology
NOX1	p22^phox^, NOXO1, NoxA1, Rac1, PDI, TKS4/5*	Caveolae on the plasma membrane, redoxisomes	Colon epithelia VSMCs, endothelial cells, uterus, placenta, prostate, osteoclasts, retinal pericytes, neurons, astrocytes, microglia	Colon cancer, prostate cancer, gastrointestinal inflammation, hypertension, restenosis after angioplasty
NOX2	p22^phox^, p67^phox^, p40^phox^, p47^phox^, Rac1/2	Phagosomes, cytoskeleton, lamellipodia, redoxisomes	Phagocytes, CNS, endothelium, VSMCs, fibroblasts, cardiomyocytes, skeletal muscle, hepatocytes, hematopoietic stem cells	Gastrointestinal inflammation, hypertension, myocardial injury, restenosis after angioplasty, melanoma, diabetes, neurodegenerative diseases
NOX3	p22^phox^, NOXO1	Plasma membrane	Inner ear, lung endothelial cells, fetal tissues	Hearing loss, pancreatic cancer
NOX4	p22^phox^, PDI, TKS4/5, Poldip2*	Focal adhesions, nucleus, endoplasmic reticulum, mitochondria	Ubiquitously expressed but highly in the kidney	Pancreatic cancer, melanoma, diabetes
NOX5	Ca^2+^, Hsp90, CaM^#^	Internal membranes, plasma membrane	Lymphatic tissue, testis, VSMCs, endothelial cells, spleen, uterus, and prostate	Atherosclerosis prostate cancer, pancreatic cancer
Duox1	Ca^2+^, DuoxA1	Plasma membrane	Thyroid, respiratory epithelium	Thyroid dysfunction, cystic fibrosis
Duox2	Ca^2+^, DuoxA2	Plasma membrane	Airway epithelial, colon, salivary gland	Thyroid dysfunction, cystic fibrosis

*Recently, the protein polymerase (DNA-directed) delta-interacting protein 2 (Poldip2) was identified to bind and to increase the activity of NOX4. Further, protein disulfide isomerase (PDI) and a p47phox analogue tyrosine kinase substrate with 4/5 SH3 domains (Tks4/5) have been reported to bind and activate NOX1 and NOX4. NOX4 is the only isoform that produces hydrogen peroxide instead of superoxide.

^#^The NOX5 protein contains four N-terminal calcium-binding sites that regulate activation of the enzyme. Activity of NOX5 can be further supported by the binding of Hsp90 or Calmodulin to the C-terminus of the protein.

**Table 2:** NOX inhibitors.

Name and origin	Mechanism of action	NOX isoform selectivity	Other pharmacological effects	References
AEBSF synthetic	Inhibits p47^phox^ assembly with oxidase subunit	NOX2	Proteases inhibitor	[64]
Apocynin picrorhiza kurroa	Inhibits p47^phox^ assembly with membrane	NOX2	H_2_O_2_ scavenging	[65]
Berberine Berberis	Inhibition of gp91^phox ^expression	NOX2	Enhancement of SOD activity	[72]
Blueberry derived polyphenols	Disrupts NOX assembly in lipid rafts	NOX2	Minimal if any ROS scavenging capacity	[82]
Celastrol Tripterygium wilfordii	Inhibition of association between cytosolic subunits and the membrane subunit	Mostly Nox1 and NOX2	None reported	[96]
DPI	Flavoprotein inhibitor	No selectivity	Inhibits NOS, xanthine oxidase, NADH ubiquinone oxidoreductase, NADH dehydrogenase, cytocrome P450 oxidoreducatese	[60–63]
EGCG green tea	Inhibits the expression of NADPH oxidase subunits	No selectivity	ROS scavenging capacity and ENOX proteins function as terminal oxidases of plasma membrane electron transport (PMET)	[87]
Emodin and rhein Rhubarb	NADPH oxidase p47^phox^ activation	NOX2?	Interfere with electron transport process and in altering cellular redox status	[74]
Ginko biloba	Inhibition of Rac1- and p47^phox^-mediated NADPH oxidase activation	NOX2?	Increases the expression of Cu-Zn superoxide dismutase heat shock protein 70	[79]
HDMPPA Fruits and nuts kimchi	Downregulates expression of p47^phox^ and Rac1	NOX2?	Preservation of NO bioavailability	[75]
Magnolol and honokiol magnolia	Inhibit ERK pathway	unknown	Inhibit NO production	[81]
Plumbagin	Unknown	Nox4	ROS scavenger	[90–95]
Prodigiosin microbial pigment	Inhibits the binding of p47^phox^ and Rac to the membrane components	NOX2?	Reduces gp91(phox) and iNOS expression	[82–84]
Resveratrol red wine	Decreases NADPH oxidase expression (p47^phox^)	NOX2?	Free-radicals-scavenging	[88, 89]
S17834	Unknown	NOX2 NOX4	None reported	[90]
Sinomenine Sinomenium acutum	Inhibits p47^phox ^translocation to the cell membrane	NOX2?	Minimal interaction with opiate receptors	[85]
